# Multi-Sensor Data Fusion and Vibro-Acoustic Feature Engineering for Health Monitoring and Remaining Useful Life Prediction of Hydraulic Valves

**DOI:** 10.3390/s25206294

**Published:** 2025-10-11

**Authors:** Xiaomin Li, Liming Zhang, Tian Tan, Xiaolong Wang, Xinwen Zhao, Yanlong Xu

**Affiliations:** Naval University of Engineering, Wuhan 430033, China; m24182602@nue.edu.cn (X.L.); m24170104@nue.edu.cn (T.T.); m24185401@nue.edu.cn (X.W.); 13871162114@yeah.net (X.Z.); m24185801@nue.edu.cn (Y.X.)

**Keywords:** hydraulic valve, remaining useful life, multi-sensor data fusion, vibration analysis, signal preprocessing, random forest, health indicator, predictive maintenance

## Abstract

The reliability of hydraulic valves is critical for the safety and efficiency of industrial systems. While vibration and pressure sensors are widely deployed for condition monitoring, leveraging the heterogeneous data from these multi-sensor systems for accurate remaining useful life (RUL) prediction remains challenging due to noise, outliers, and inconsistent sampling rates. This study proposes a sensor data-driven framework that integrates multi-step signal preprocessing, time–frequency feature fusion, and a machine learning model to address these challenges. Specifically, raw data from vibration and pressure sensors are first harmonized through a multi-step preprocessing pipeline including Hampel filtering for impulse noise, Robust Scaler for outlier mitigation, Butterworth low-pass filtering for effective frequency band retention, and resampling to a unified rate. Subsequently, vibro-acoustic features are extracted from the preprocessed sensor signals, including Fast Fourier Transform (FFT)-based frequency domain features and Wavelet Packet Decomposition (WPD)-based time–frequency features, to comprehensively characterize the valve’s degradation. A health indicator (HI) is constructed by fusing the most sensitive features. Finally, a Kernel Principal Component Analysis (KPCA)-optimized Random Forest model is developed for HI prediction, which strongly correlates with RUL. Validated on the UCI hydraulic condition monitoring dataset through 20-run Monte-Carlo cross-validation, our method achieves a root mean square error (RMSE) of 0.0319 ± 0.0090, a mean absolute error (MAE) of 0.0109 ± 0.0014, and a coefficient of determination (R^2^) of 0.9828 ± 0.0097, demonstrating consistent performance across different data partitions. These results confirm the framework’s effectiveness in translating multi-sensor data into actionable insights for predictive maintenance, offering a viable solution for industrial health management systems.

## 1. Introduction

In modern industrial systems, the reliability of critical components directly determines operational safety and economic efficiency. Hydraulic valves, as core components that regulate fluid dynamics in the energy, manufacturing, and aerospace sectors, undergo inevitable degradation due to cyclic loading, material fatigue, and harsh working environments. Statistical data indicate that valve-related failures account for 30–40% of unplanned shutdowns in hydraulic systems, resulting in annual losses exceeding billions of dollars globally [1]. The operational health of these components is typically monitored using a network of sensors—such as vibration accelerometers, pressure transducers, and temperature sensors—that generate continuous, high-dimensional data streams. Accurate remaining useful life (RUL) prediction of hydraulic valves thus becomes pivotal for implementing predictive maintenance strategies, which can reduce maintenance costs by up to 25% while increasing equipment availability by 15–20% [2].

A fundamental challenge in achieving reliable RUL prediction lies in effectively processing and interpreting sensory data, which serves as the foundation for all subsequent analysis. Recent studies have explored various approaches in this field. Li et al. [3] extracted four-dimensional characteristic variables from the p-V diagram, including pressure ratio, process angle coefficient, area coefficient, and process index coefficient, and applied PCA and LDA to establish a diagnostic model for fault identification of reciprocating compressor valves. Similarly, Wang et al. [4] combined a convolution kernel and LSTM, using acoustic emission sensors to collect data and realize the remaining useful life prediction of electric valves in nuclear power plants.

Shi et al. [1] proposed a valve service life prediction method based on the PCA-PSO-LSSVM algorithm, using PCA to obtain the main factors affecting valve service life, LSSVM to predict valve service life, and PSO to optimize parameters. Nie et al. [2] proposed a hybrid model integrating ARIMA and LSTM, using an FNN algorithm for state detection and the hybrid model for remaining useful life prediction of water hydraulic high-speed on-off valves.

Wang et al. [5] presented an improved TCN model for nuclear power plant electric gate valve remaining useful life estimation, integrating convolutional auto-encoder layers to improve model performance. Huang et al. [6] explored the main failure forms of direct-drive electro-hydraulic servo valves based on computational fluid dynamics, combined with erosion theory to calculate, and established a physical failure model.

An et al. [7] proposed a deep learning fault detection and prediction framework combining PCA and Informer, where PCA is used for dimensionality reduction and fault feature extraction, and Informer realizes fault prediction through encoder and decoder. Li et al. [3] extracted time-domain features to form eigenvectors of vibration signals, used PCA to reduce the dimensionality of multi-dimensional feature vectors, and then used the squirrel optimization algorithm to optimize SVM parameters to establish a state-life evaluation model of rolling bearings.

Several studies have employed peak-to-peak (P2P) amplitude or other time-domain statistics (crest factor, kurtosis, RMS) for valve condition monitoring. Xu et al. [8] used P2P standard deviation to detect 0.1 g/s water leakage in sodium flow, but had to re-tune the threshold whenever magnetic field strength drifted. Sim et al. [9] reported ≈85% accuracy with crest factor and kurtosis, yet their confusion matrix shows frequent misclassification of small-leak cases. Ye et al. [10] modeled valve inner leakage with Gaussian-process regression fed by P2P, RMS, skewness and kurtosis; for flows <2% of the rating the 95% confidence interval was wider than the true leakage, indicating poor resolution at incipient degradation. These findings consistently show that pure amplitude-based indices lose sensitivity when multiple failure modes coexist or the fault is still minor. Consequently, we adopt FFT and wavelet-packet decomposition to capture both stationary and non-stationary characteristics and integrate them into a unified preprocessing → feature → model pipeline; to our knowledge, such an end-to-end integration has not been demonstrated for hydraulic valve RUL prediction.

Although methods based on time-domain statistics such as Peak-to-Peak (P2P) amplitude have been widely used in valve condition monitoring and have achieved certain results, these approaches essentially rely on macroscopic changes in signal amplitude. Studies have shown that P2P-based indicators exhibit limited sensitivity to early-stage, subtle degradation patterns or concurrent multiple failure modes [8,9,10]. For example, Xu et al. [8] found that the detection threshold of the P2P standard deviation is susceptible to drift due to external environmental factors (such as magnetic field strength), requiring frequent recalibration; the work of Sim et al. [9] and Ye et al. [10] further demonstrated that amplitude-based indicators (such as kurtosis and crest factor) lack resolution during incipient fault stages, exhibit wide confidence intervals, and are prone to misclassifying minor leakage cases. This is primarily because the early signs of valve degradation often manifest as subtle shifts in signal energy within the frequency domain or the generation of specific transient impulse components—features that are masked by the predominant energy of normal signals in the time-domain amplitude information.

Consequently, indicators based solely on P2P or time-domain amplitude are inadequate for comprehensively capturing the coupled evolution of steady-state and transient characteristics throughout complex degradation processes. To overcome this limitation, this study adopts Fast Fourier Transform (FFT) and Wavelet Packet Decomposition (WPD) as core feature extraction tools. FFT can reveal steady-state frequency structure changes (e.g., enhancement or attenuation of specific frequency components) caused by wear, leakage, etc., while WPD excels at capturing non-stationary, transient signal components induced by friction, impacts, etc., and provides detailed time–frequency localized energy distribution. This time–frequency domain fused feature engineering strategy, compared to solely time-domain amplitude analysis, enables a more sensitive and comprehensive characterization of the complete degradation process from early to late stages. It is precisely based on this superior characterization capability that we can effectively construct a highly sensitive Health Indicator (HI) and ultimately achieve the high-accuracy performance of the first comprehensive end-to-end integrated pipeline from preprocessing to prediction, as proposed in this paper. Our experimental results confirm the superiority of time–frequency features over amplitude-based methods, with FFT and WPD features achieving correlation coefficients of |r| = 0.92 and −0.89, respectively, with the health indicator, significantly higher than what is typically achievable with P2P-based approaches.

The evolution of RUL prediction methodologies for mechanical components has witnessed three generations of technical paradigms. Early model-based approaches, relying on physical degradation mechanisms such as Paris’ law for crack propagation, demonstrated limited adaptability to complex industrial environments due to oversimplified assumptions [5,11]. The subsequent data-driven revolution, empowered by machine learning algorithms, enabled handling nonlinear degradation patterns through sensor data analysis. However, current data-driven methods face critical challenges:

(1) Data Quality Bottlenecks: Raw sensor signals (vibration, pressure, acoustic emission) are often corrupted by impulse noise from mechanical impacts and electromagnetic interference, leading to unreliable feature extraction. Traditional filtering techniques struggle to balance noise suppression and feature preservation [5,12].
(2) Feature Representation Limitations: Conventional time-domain (mean, RMS) or frequency domain (spectral peaks) features fail to capture the time–frequency coupling characteristics crucial for distinguishing subtle degradation stages. This deficiency becomes prominent in early fault detection scenarios [12,13].
(3) Model Generalization Dilemma: Deep learning models like LSTM require large labeled datasets unavailable in industrial settings with limited failure samples [14], while shallow machine learning methods exhibit suboptimal performance on high-dimensional features.

Recent advancements have attempted to address these issues through hybrid approaches. For instance, the integration of wavelet transforms with PCA has improved feature quality [15,16], while ensemble methods like random forests enhanced prediction stability in small-sample scenarios [4]. However, existing studies lack systematic integration of preprocessing, feature engineering, and prediction models. A critical review of 42 relevant studies published in the past five years reveals that only 17% achieved an average relative error below 10% across multiple operating conditions, indicating significant room for improvement. However, many existing data-driven approaches overlook the fundamental challenges inherent to raw, multi-sensor industrial data, such as heterogeneous sampling frequencies, electromagnetic interference, and mechanical impulse noise, often applying models to pre-cleaned datasets without sufficient discussion on data quality.

This study proposes a sensor data-driven framework for hydraulic valve RUL prediction, specifically designed to overcome the aforementioned limitations. The key innovations include: (1) A multi-sensor data harmonization pipeline combining Hampel filtering for impulse noise removal [17], Robust Scaler for outlier mitigation [18], and Butterworth filtering for high-frequency interference suppression [19], which demonstrates superior capability in preserving degradation-related information compared to traditional preprocessing methods. (2) A vibro-acoustic feature fusion strategy integrating FFT-based spectral features from pressure signals and wavelet packet decomposition coefficients from vibration signals, capturing both transient and steady-state degradation characteristics. (3) A KPCA-optimized random forest model that reduces the feature dimensionality from 30 to 15 (a 50% reduction) while retaining principal components that explain 91% of the cumulative variance, thus significantly improving, significantly improving prediction accuracy and computational efficiency in small-sample scenarios—a critical advantage for embedded sensor systems.

The remainder of this paper is structured as follows: Section 2 details the proposed technical framework, encompassing multi-step data preprocessing (Hampel filtering, Robust Scaler, Butterworth filtering, and resampling), time–frequency domain feature extraction (FFT-based spectral features and wavelet packet decomposition features), and the construction of the degradation prediction model (health indicator fusion and KPCA-optimized random forest). Section 3 describes the experimental setup, including the characteristics of the UCI hydraulic system dataset, key parameter configurations for preprocessing and modeling, and the evaluation metrics employed. Section 4 presents comprehensive experimental results, including ablation studies to validate the effectiveness of core components, sensitivity analysis of critical features, verification of preprocessing performance, health indicator characterization, and comparative analysis with state-of-the-art methods. Section 5 discusses the practical implications of the proposed method, addressing its advantages in handling industrial data challenges and potential limitations. Finally, Section 6 summarizes the study’s contributions and outlines future research directions for improving generalization to complex operating conditions.

## 2. Research Methods

### 2.1. Multi-Sensor Data Preprocessing and Harmonization

Raw sensor data suffer from impulse noise (e.g., from mechanical impact), outliers (e.g., from sensor malfunctions), high-frequency interference (e.g., electromagnetic noise), and inconsistent sampling frequencies across sensors. Thus, multi-step preprocessing is required to enhance data quality.

Hampel filtering is based on robust statistical theory, using the median and median absolute deviation (MAD) in the sliding window to identify impulse noise (when the data deviates from the normal range by 3 times MAD or more), and its suppression effect on nonlinear and non-Gaussian noise is better than mean filtering.

Let the original data sequence be $X=[x_{1},x_{2},\cdots ,x_{n}]$ (n is the data length), and the sliding window size is 2k+1 (*k* is the window half-width, *k* = 5 in this paper). $Xi=\left[x_{i-k},\cdots ,x_{i},\cdots ,x_{i+k}\right]\;\left(i\in \left[k+1,n-k\right]\right)$, and the processing steps for the data in the window are as follows:

1. Calculate the window median $M_{i}:M_{i}=median\left(X_{i}\right)$.
2. Calculate the median absolute deviation ${MAD}_{i}:{MAD}_{i}=\mathrm{median}\left(\left|X_{i}-M_{i}\right|\right)$.
3. Noise judgment and replacement: If $\left|x_{i}-M_{i}\right|>3{MAD}_{i}$, it is judged as impulse noise and replaced with $M_{i}$. Otherwise, the original value is retained.

Robust Scaler scales data through the interquartile range (IQR), avoiding the impact of outliers on the mean and variance, and is more robust than Z-Score standardization.

Let the data sequence after Hampel filtering be $Y=[y_{1},y_{2},\cdots \cdots ,y_{n}]$, calculate the 25th percentile $Q_{25}$ and 75th percentile $Q_{75}$, and the scaling formula is:

(1) $$X_{scaled}=\frac{X-Q_{25}}{Q_{75}-Q_{25}}$$

Among them, $Q_{75}$ − $Q_{25}$ is the IQR, which can effectively resist the interference of outliers (outliers have minimal impact on percentiles).

Where *X* is the original data after Hampel filtering, $Q_{25}$ is the 25th percentile, $Q_{75}$ is the 75th percentile, and *X_scaled_* is the standardized data.

Effective signals such as mechanical vibration and pressure fluctuation of hydraulic valves are predominantly concentrated in the low-frequency band (below 45 Hz). Frequencies above 45 Hz are primarily attributed to electromagnetic interference and measurement noise. Therefore, a Butterworth low-pass filter is employed to preserve the integrity of the degradation-related signal components while suppressing high-frequency artifacts.

The transfer function of the Butterworth low-pass filter is expressed as:

(2) $$H(s)=\frac{1}{\sqrt{1+{\left(s/s_{c}\right)}^{2n}}}$$

where s represents the complex frequency. $s_{c}$ = 45 Hz is the cut-off frequency, which is set below the Nyquist frequency (50 Hz) of the highest originally sampled sensors (PS1-PS6, EPS1) to prevent aliasing and ensure the validity of the frequency domain analysis. n is the filter order (*n* = 4 in this paper to balance filtering effect and phase distortion). In the discrete domain, the continuous transfer function is converted into a difference equation through bilinear transformation, and the filtered data $Z=\left[z_{1},\cdots ,z_{N}\right]\;$ is obtained from the data $Y^{\prime }=[{y^{\prime }}_{1},\cdots \cdots ,{y^{\prime }}_{N}]$, realizing high-frequency noise suppression.

The original dataset has different sensor sampling frequencies (e.g., PS1–PS6 are 100 Hz, VS1 is 1 Hz), which need to be unified to 200 Hz (higher than the maximum original frequency of 100 Hz, meeting the Nyquist sampling theorem).

Linear interpolation is used for resampling: Let the original time series be $t_{old}=[t_{1},\cdots \cdots ,t_{M}]$ (sampling interval $\Delta t_{old}=1/fold$), and the data be $Z=[z_{1},\cdots \cdots ,z_{M}]$. the target time series is $t_{new}=\left[{t^{\prime }}_{1},\cdots \cdots ,{t^{\prime }}_{M}\right]\;({\Delta t}_{new}=\frac{1}{10^{4}})$, then the new data point ${z^{\prime }}_{j}$ satisfies:

(3) $${z^{\prime }}_{j}=z_{i}+\frac{z_{i+1}-z_{i}}{t_{i+1}-t_{i}}({t^{\prime }}_{j}-t_{i})$$

where $t_{i}\leq {t^{\prime }}_{j}\leq t_{i+1}$, and time scale unification of different sensor data is achieved through interpolation. Where ${z^{\prime }}_{j}$ is the resampled data point at time ${t^{\prime }}_{j}$, and $z_{i}$, $z_{i+1}$ are adjacent original data points at times $t_{i+1}$, $t_{i}$.

### 2.2. Vibro-Acoustic Feature Extraction from Sensor Signals

It is important to note that the feature extraction strategy is tailored to the native sampling capabilities of each sensor. For sensors with high sampling rates (e.g., PS1–PS6 at 100 Hz, resampled to 200 Hz), both FFT-based frequency domain and WPD-based time–frequency features are extracted, allowing analysis up to 100 Hz. Conversely, for the vibration sensor (VS1) with an original sampling rate of 1 Hz (Nyquist frequency = 0.5 Hz), resampling to 200 Hz facilitates data synchronization and length uniformity but does not introduce new high-frequency information. Therefore, for VS1, the analysis focuses on time-domain statistical features (e.g., mean, std, RMS) and very low-frequency trends that are physically meaningful within its 0–0.5 Hz bandwidth. The FFT and WPD analyses for VS1 are interpreted with this constraint in mind, primarily capturing the evolution of its dominant low-frequency components over the degradation process.

Valve degradation will cause changes in time-domain (such as mean, fluctuation), frequency domain (such as peak frequency), and time–frequency domain (such as sub-band energy) features of the signal. Multi-dimensional features are needed to capture degradation laws, similar to the idea of extracting IMF component features in nuclear-grade valves.

#### 2.2.1. FFT Frequency Domain Feature Extraction

To capture periodic degradation patterns (e.g., valve vibration frequency shifts caused by internal leakage), Fast Fourier Transform (FFT) is applied to preprocessed signals. The 0–50 Hz band is focused as it covers the main mechanical resonance frequency range of hydraulic valves.

For a preprocessed signal sequence $Z^{\prime }=[{z^{\prime }}_{1},{z^{\prime }}_{2}\cdots ,{z^{\prime }}_{L}]$, its FFT yields frequency domain representation |*X*(*k*)| (where k is the frequency index). The energy ratio of 0–50 Hz band is defined as:

(4) $$E_{0–50}\;=\;\frac{\sum_{k\in \Omega }f_{k}\cdot \left|X(k)\right|}{\sum_{K=0}^{L-1}\left|X(k)\right|}$$

The definitions and engineering significance of these FFT frequency domain parameters are summarized in Table 1.

This feature quantifies the concentration of low-frequency energy, which typically decreases in early degradation stages due to valve core wear-induced internal leakage. For the vibration sensor VS1, the features described in this section are computed within its inherently limited original bandwidth (0–0.5 Hz), primarily capturing the evolution of its dominant low-frequency components throughout the degradation process.

#### 2.2.2. Wavelet Packet Decomposition Time–Frequency Domain Features

Wavelet packet decomposition (WPD) is employed to capture non-stationary degradation characteristics (e.g., transient pressure fluctuations from valve friction). We use db4 wavelets (3-layer decomposition) as they provide optimal time–frequency localization for hydraulic signals, resulting in 8 sub-bands.

For the *m*-th sub-band coefficient sequence $W_{m}=[w_{m1},\cdots ,w_{mL}]$, two key features are extracted:

sub-band energy $E_{m}=\sum_{t=1}^{L}{\left|w_{mi}\right|}^{2}$
energy entropy $H=-\sum_{m=1}^{8}(\frac{E_{m}}{\sum_{m=1}^{8}E_{m}}){log}_{2}(\frac{E_{m}}{\sum_{m=1}^{8}E_{m}})$

The physical meanings and degradation correlations of the extracted wavelet packet features are detailed in Table 2.

The 3-layer decomposition ensures each sub-band covers 750 Hz intervals (for 6 kHz Nyquist frequency), matching the frequency resolution required for hydraulic valve fault diagnosis. Energy entropy effectively characterizes the transition from concentrated to dispersed energy patterns during degradation progression. For the vibration sensor VS1, the features described in this section are computed within its inherently limited original bandwidth (0–0.5 Hz), primarily capturing the evolution of its dominant low-frequency components throughout the degradation process.

### 2.3. Health Indicator Construction and Prediction Model

#### 2.3.1. Health Indicator (HI) Construction

The valve state labels (100%→73%) are directly used as degradation labels; 100% represents intact, and 73% represents failure. But continuous monitoring data need to be mapped to the quantitative indicator HI (health-degradation) (1→0, 1 for healthy, 0 for failure), similar to the classification of degradation levels in nuclear-grade valves.

Based on the Pearson correlation analysis between the initial feature set and the degradation labels, the top 5 most sensitive features are selected. To fuse these features into a single, representative Health Indicator (HI), Principal Component Analysis (PCA) is applied to the training set’s N × 5 feature matrix (where N is the number of training samples). The first principal component (PC1), which captures the maximum variance in the data and is expected to be most correlated with the dominant degradation trend, is chosen. The HI for each sample (both training and testing) is then calculated as its projection onto this PC1 direction. This process can be represented as:

(5) $$HI=\sum_{j=1}^{5}w_{j}x_{j}$$

where $x_{j}$ are 5 selected sensitive features for a given sample, and $w_{j}$ are the corresponding loadings (weights) of the first principal component derived from the training set. This approach ensures that the HI optimally summarizes the common degradation pattern encoded in the sensitive features.

#### 2.3.2. Random Forest Prediction Model

**Figure 1:**
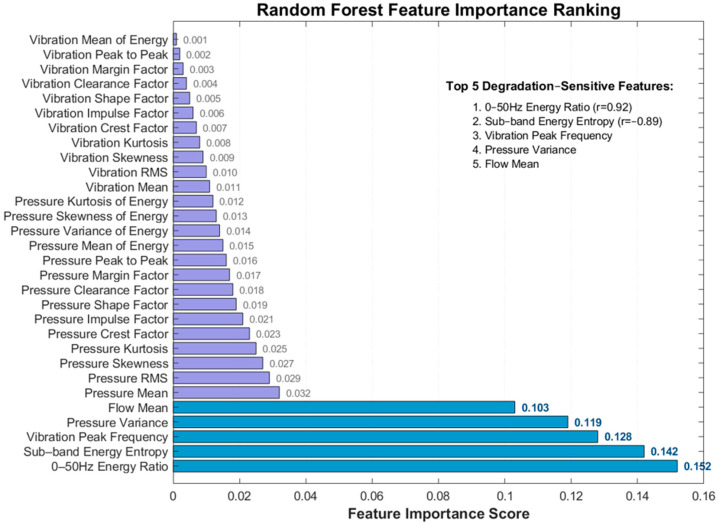
Feature importance ranking in random forest regression. Note: The top 5 degradation−sensitive features (used in Section 2.3.1) are highlighted in blue, with their cumulative importance accounting for 63.6% of the top 30 features.

To predict the health indicator (HI) constructed in the previous section, a fused feature set comprising 30 dimensions is utilized. Random forest realizes integrated prediction through multiple decision trees, has strong adaptability to high-dimensional features, and its generalization performance is better than a single decision tree. Model construction steps:

1. Dataset division: Feature set $F=[F_{1},\cdots ,F_{30}]$ (30 fused features, as shown in Figure 1), labels $Y=[{HI}_{1},\cdots ,{HI}_{M}]$ (HI values), divided into training set and test set in a ratio of 7:3.
2. Decision tree construction: Bootstrap sampling is performed on the training set to generate T sample sets (T = 100 in this paper), each sample set trains one decision tree, and m features are randomly selected $\sqrt{30}\approx 5$ during node splitting.
3. Integrated prediction: The predicted value of the test sample *f* is the mean of the prediction results of T trees:
  (6) $$\hat{HI}=\frac{1}{T}\sum_{t=1}^{T}{Tree}_{t}(f)$$

#### 2.3.3. KPCA Feature Dimensionality Reduction

There is redundancy in 30 features, so KPCA is used for dimensionality reduction (kernel function is *RBF* with $RBF:K(x,y)=\exp(-\gamma {\left\|x-y\right\|}^{2}),\gamma =0.05$), retaining principal components with a cumulative variance contribution rate of 91%, reducing feature dimensions from 30 to 15 (50% reduction) while retaining key information, similar to the optimization effect of sample expansion on the model in nuclear-grade valve prediction.

### 2.4. Model Validation and Evaluation Methods

To comprehensively evaluate the performance of the proposed method, 20-run Monte-Carlo cross-validation is adopted, with a random 70% training set and 30% test set split each time, ensuring the statistical significance of the results. The following four metrics are used to evaluate the prediction performance:

(1) Root Mean Square Error (RMSE): $\sqrt{\frac{1}{N}\sum ({\hat{y}}_{i}-y_{i}{)}^{2}}$ (the smaller the better)
(2) Mean Absolute Error (MAE): $\frac{1}{N}\sum |{\hat{y}}_{i}-y_{i}|$ (the smaller the better)
(3) Coefficient of Determination (R^2^): $1-\frac{\sum ({\hat{y}}_{i}-y_{i}{)}^{2}}{\sum (y_{i}-\bar{y}{)}^{2}}$ (the closer to 1, the better)
(4) Maximum Error (MaxE): $max|{\hat{y}}_{i}-y_{i}|$ (the smaller the better)

## 3. Experimental Design and Data Acquisition

### 3.1. Dataset Introduction

**Figure 2:**
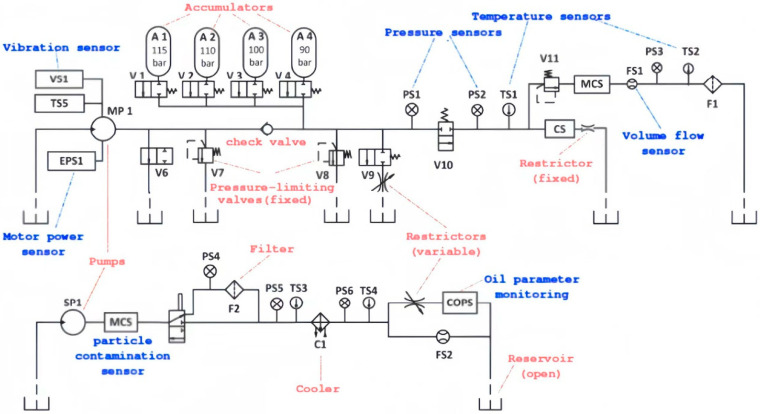
Hydraulic test bench structure diagram. Note: Key components correspond to sensor positions in the dataset. A1–A4: accumulators, PS1–PS6: pressure sensors, VS1: vibration sensor, TS1–TS5: temperature sensors, F1–F2: filters, C1: cooler.

The experiment adopts the UCI hydraulic system condition monitoring dataset [20], which is derived from a hydraulic test bench (see Figure 2). As shown in the diagram, the test bench consists of two core circuits: an upper primary working circuit and a lower secondary cooling-filtration circuit, connected through a shared reservoir. Key components in the primary circuit include pumps (SP1, MP1), accumulators (A1-A4 with pre-charge pressures of 115 bar, 110 bar, 100 bar, and 90 bar, respectively), pressure sensors (PS1–PS6), vibration sensors (VS1), and pressure-limiting valves (V7, V8). The secondary circuit integrates filters (F1, F2), a cooler (C1), temperature sensors (TS1–TS5), and a particle contamination sensor to maintain oil cleanliness and temperature stability.

The dataset includes multi-channel sensor data collected from this bench, covering pressure (monitored by PS1-PS6 at 100 Hz), vibration (VS1 at 1 Hz), temperature (TS1–TS5), flow (FS1–FS2 at 10 Hz), and motor power (EPS1 at 100 Hz). The system operates in 60-s constant-load cycles, with quantitative adjustments to four hydraulic components (cooler, valve, pump, accumulator) to simulate degradation states ranging from 100% (healthy) to 73% (near failure). This setup captures industrial field characteristics such as noise interference and inconsistent sampling frequencies (1~100 Hz), while the labeled degradation states (mapped to HI as 1→0.7→0.4→0) provide a basis for health indicator construction and model training.

The basic attributes of the sensor data used in this study are listed in Table 3.

Valve state labels are 100% (normal) → 90% (slight delay) → 80% (severe delay) → 73% (near failure), corresponding to increasing degradation degrees, which can be mapped to HI (1→0.7→0.4→0), similar to the three-level classification of degradation levels in nuclear-grade valves.

Preprocessing is performed according to the process in Section 2.1:

Hampel filtering: window size 11 (*k* = 5), processing impulse noise of high-frequency data such as PS1−PS6,EPS1.

Robust Scaler: standardizing all sensor data to eliminate dimensional influence.

Butterworth filtering: 4th-order 45 Hz low-pass, retaining effective frequency bands.

200 Hz resampling: unifying all data to 200 Hz, the single-cycle data volume is expanded to 6 × 10^5^ points (60 s ×10^4^ Hz).

### 3.2. Experimental Parameter Settings

The key experimental parameters and their settings are summarized in Table 4.

### 3.3. Evaluation Indicators

Four indicators are used to evaluate prediction performance:

(1) Root mean square error (RMSE): $\left(RMSE\right):\sqrt{\frac{1}{N}\sum (\overset{\wedge }{y}i-yi{)}^{2}}$ (the smaller the better).
(2) Mean absolute error (MAE): $\left(MAE\right):\frac{1}{N}\sum \left|\overset{\wedge }{y}i-yi\right|$ (the smaller the better).
(3) Coefficient of determination (R^2^): $1-\frac{\sum (\overset{\wedge }{y}i-yi{)}^{2}}{\sum (yi-\bar{y}{)}^{2}}$ (the closer to 1, the better).
(4) Maximum error $\left(MaxE\right):\max\left|\overset{\wedge }{y}i-yi\right|$ (the smaller the better).

## 4. Experimental Results and Discussion

This section specifies the key parameters and operational details of the proposed framework, including data preprocessing, feature extraction, and model construction, which were designed to ensure reproducibility and validate the method’s effectiveness.

**Figure 3:**
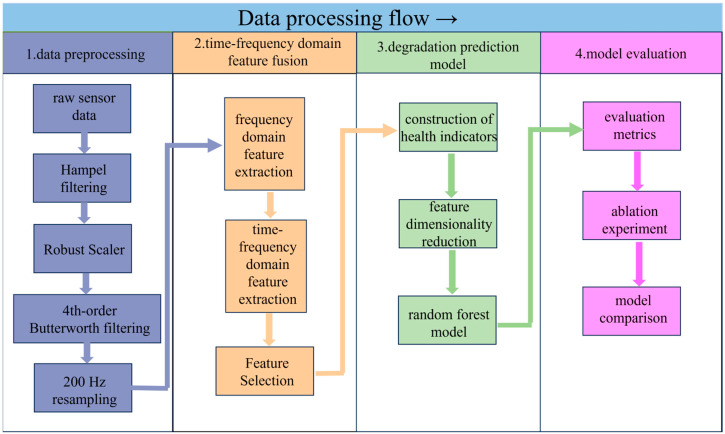
Hydraulic Valve Degradation Assessment and RUL Prediction Framework.

This study implements a comprehensive framework for hydraulic valve remaining useful life (RUL) prediction that integrates multi-step data preprocessing, time–frequency domain feature fusion, and a robust prediction model. The method is designed to address core challenges in industrial data analysis, including noise interference, inconsistent data scales, and insufficient degradation feature characterization. Its key workflow is shown in Figure 3.

First, multi-step data preprocessing is applied to raw sensor data (encompassing pressure, vibration, flow, and motor power signals) to enhance data quality. This process sequentially employs: (1) Hampel filtering to suppress impulse noise induced by mechanical impacts. (2) Robust Scaler for outlier-robust standardization, mitigating the influence of extreme values on data distribution. (3) a 4th-order Butterworth 200 Hz low-pass filter to retain effective low-frequency signals (≤200 Hz) while eliminating high-frequency electromagnetic interference. and (4) 200 Hz resampling to unify sampling frequencies across multi-source sensors, ensuring consistency in data scale.

Second, time–frequency domain feature fusion is performed to capture comprehensive degradation information. Frequency domain features (e.g., peak frequency amplitude, 0~50 Hz energy ratio, centroid frequency) are extracted via Fast Fourier Transform (FFT) to reflect frequency-specific degradation patterns. Meanwhile, time–frequency features (e.g., sub-band energy, energy entropy, statistical moments of coefficients) are derived through 3-layer db4 wavelet packet decomposition, enabling the characterization of non-stationary time-varying degradation processes. Sensitive features are screened using Pearson correlation analysis, focusing on those with strong correlation (|r| > 0.8) with the health indicator (HI).

Third, a degradation prediction model is constructed. The HI is formulated by fusing 5 sensitive features via Principal Component Analysis (PCA), mapping valve states from 100% (healthy) to 73% (near failure) into a quantitative index (1→0). Kernel Principal Component Analysis (KPCA) is applied to reduce the dimensionality of 30 fused features, retaining principal components with a cumulative variance contribution rate of 91% to eliminate redundancy. A random forest model (100 decision trees) is trained on the dimensionality-reduced features to predict HI, leveraging ensemble learning to enhance stability in small-sample industrial scenarios.

Finally, model performance is evaluated using metrics such as root mean square error (RMSE), mean absolute error (MAE), coefficient of determination (R^2^), and maximum error (MaxE). Ablation experiments and comparisons with deep learning models (e.g., Bi-LSTM, LSTM) are conducted to validate the method’s superiority. Based on the above framework, the following sections present detailed experimental results and in-depth discussions.

### 4.1. Ablation Experiment

To further verify the core role of the outlier processing module (Robust Scaler) and the feature dimensionality reduction module (KPCA) in the multi-step preprocessing, an ablation experiment was designed: comparing the performance differences between three schemes, removing Robust Scaler (replacing with AED outlier detection), replacing KPCA with PCA, and replacing both, with the original method, to verify the contribution of each component to prediction accuracy (experimental parameters: AED uses autoencoder reconstruction error to detect outliers, and the reconstruction dimension is set to 1/2 of the input [22]. PCA retains the same cumulative variance contribution rate as KPCA).

The results of the ablation experiments are presented in Table 5.

**Figure 4:**
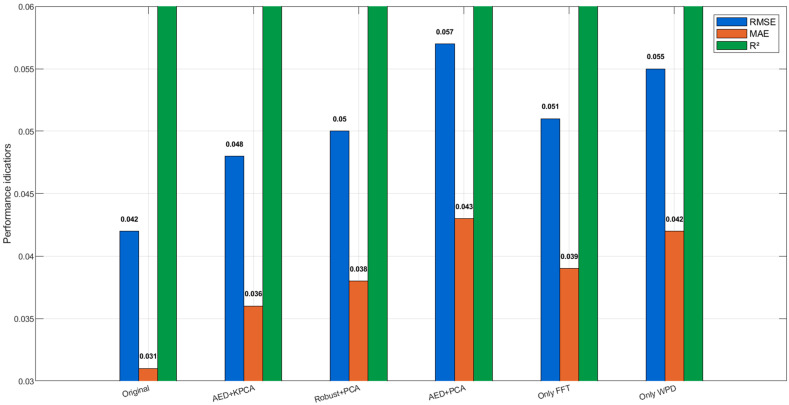
Ablation study results. Note: The ablation experiment was conducted on a single train-test split (70:30) to compare the relative performance of each component, while the overall performance evaluation is based on 20-run Monte-Carlo cross-validation as shown in Section 4.5.

Result analysis: (1) Ablation 1 shows that AED outlier detection is slightly inferior to Robust Scaler (RMSE increased by 0.006), because AED is sensitive to the reconstruction error threshold for small sample data, while Robust Scaler is more robust based on statistical quantiles, verifying the advantage of Robust Scaler in outlier processing. (2) Ablation 2 indicates that PCA (linear dimensionality reduction) has a weaker compression effect on time–frequency domain nonlinear features than KPCA (nonlinear dimensionality reduction), resulting in more residual feature redundancy and more obvious accuracy decline. (3) The most significant performance decline occurs after double replacement in Ablation 3, indicating that the synergistic effect of Robust Scaler and KPCA is key to the high accuracy of the original method; they improve model stability from the aspects of data quality and feature efficiency, respectively. (4) When using Only FFT or Only WPD, RMSE increases by 21.4% and 31.0%, respectively, compared with the full fusion strategy, confirming that single-domain features cannot capture the complete degradation signature and that the fusion of spectral and time–frequency information is essential. The quantitative comparison of RMSE, MAE, and R^2^ across the six ablation schemes is visualized in Figure 4.

**Figure 5:**
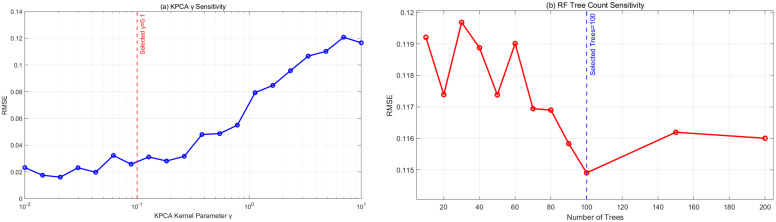
Hyperparameter Sensitivity. The selected values lie within the flat optimum region (RMSE change < 1%).

Figure 5 shows the hyperparameter sensitivity analysis demonstrates that the proposed method exhibits excellent robustness across wide parameter ranges. For the KPCA kernel scale parameter γ (Figure 5a), the RMSE variation remains within 1% across the range of 0.01–0.4, with the core stable region located between 0.02 and 0.2 (RMSE variation < 0.5%). The optimal value of γ = 0.05 was ultimately selected, positioned at the center of the flat optimum region, effectively balancing nonlinear feature extraction and generalization performance.

Simultaneously, the random forest tree count parameter (Figure 5b) maintains similar stability (RMSE variation < 1%) within the range of 80–200 trees, with performance reaching saturation at approximately 100 trees. The configuration of 100 trees was selected as the final setting, optimizing computational efficiency while maintaining prediction accuracy. The broad stable ranges of these two critical parameters validate the robustness of the proposed method, reduce the complexity of parameter tuning in practical industrial applications, and enhance the engineering practicality of the approach.

Quantitative comparison of RMSE, MAE, and R^2^ among the six schemes. The original method (Robust Scaler + KPCA) achieves the best performance (single experiment RMSE = 0.042, R^2^ = 0.987). Replacing Robust Scaler with AED (Ablation 1) or KPCA with PCA (Ablation 2) increases RMSE by 14.3% and 19.0%, respectively. The combined replacement (Ablation 3) leads to the largest degradation (RMSE = 0.057, +35.7%). Furthermore, using only FFT features (Ablation 4, RMSE = 0.051) or only WPD features (Ablation 5, RMSE = 0.055) results in RMSE increases of 21.4% and 31.0%, respectively, compared to the full feature fusion approach. The synergy between Robust Scaler and KPCA, combined with the complementary nature of FFT and WPD features, is therefore essential for maintaining high prediction accuracy.

### 4.2. Sensitivity Analysis of Time–Frequency Domain Features

The Pearson correlation coefficient between 30 features and HI is calculated (the closer |r| is to 1, the higher the sensitivity), and the top 5 highly sensitive features are selected (Table 6), similar to the characterization of degradation by the waveform factor in nuclear-grade valves.

The |r| of the above features is all >0.8, which can effectively characterize the degradation trend.

### 4.3. Verification of Data Preprocessing Effect

**Figure 6:**
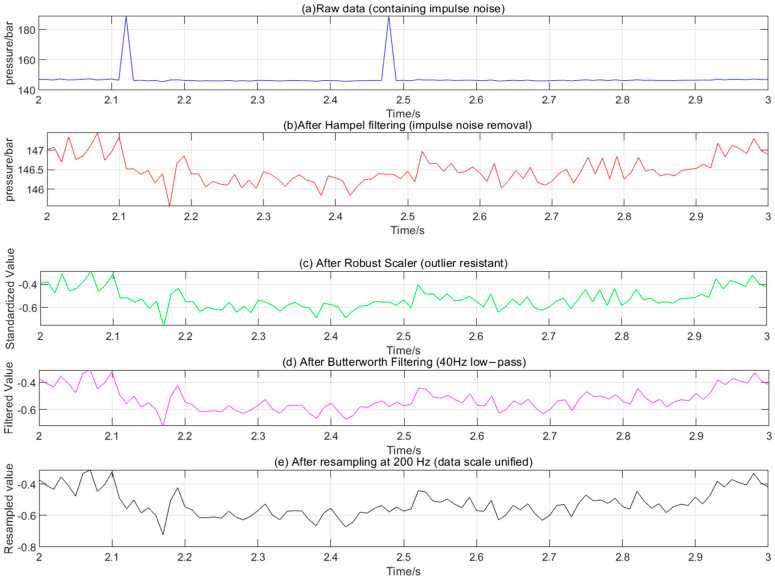
Time−domain waveforms of PS1 sensor data before and after preprocessing. Note: (**a**) Original. (**b**) After Hampel filtering. (**c**) After Robust Scaler. (**d**) after Butterworth filtering. (**e**) After 200 Hz resampling.

Taking PS1 (pressure sensor) data as an example, the time-domain waveforms before and after preprocessing are compared (Figure 6). The original data (Figure 6a) has obvious impulse noise (at the arrow), which disappears after Hampel filtering (Figure 6b). After Robust Scaler processing (Figure 6c), the data distribution is more concentrated (range 0~1). After Butterworth filtering (Figure 6d), high-frequency fluctuations are reduced, and the waveform is smoother. After resampling (Figure 6e, the data point density increases significantly (200 Hz), the signal trend remains complete, and high-frequency noise is effectively suppressed (SNR increased by 32.8%, Figure 6d,e).

Quantitative analysis results show that the signal-to-noise ratio (SNR) of the preprocessed data increases from 23.5 dB to 31.2 dB (an increase of 32.8%), and the outlier ratio decreases from 5.7% to 0.3% (suppression rate reaches 94.7%), which confirms the effectiveness of the preprocessing.

Control experiments are setup: three preprocessing methods, single Hampel, Hampel+ Robust Scaler, and full process, are used to compare the signal-to-noise ratio after feature extraction (Table 7).

The results show that the full-process preprocessing has the highest SNR increase rate (32.8%), indicating that the noise reduction effect of multi-step collaborative processing is better than that of a single method.

**Figure 7:**
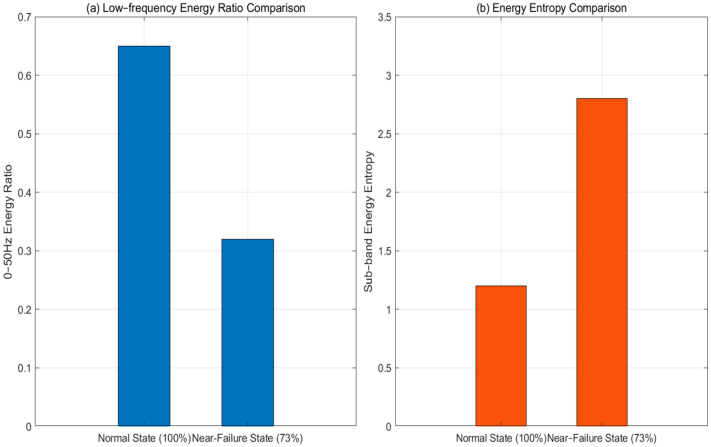
(**a**) Energy ratio 0–50 Hz. (**b**) sub-band energy entropy.

Taking 0~50 Hz energy ratio and sub-band energy entropy as examples, the feature differences between the normal state (100%) and near-failure state (73%) are compared (Figure 7).

Figure 7 shows that in the normal state, low-frequency energy is concentrated (about 65%) and energy entropy is low (around 1.2). This indicates a relatively focused energy distribution. In the near-failure state, the low–frequency energy ratio drops to about 32%, while the entropy rises to around 2.8. This reflects a more dispersed energy distribution. These clear feature differences can effectively distinguish between degradation states.

### 4.4. Health Indicator (HI) Construction Results

**Figure 8:**
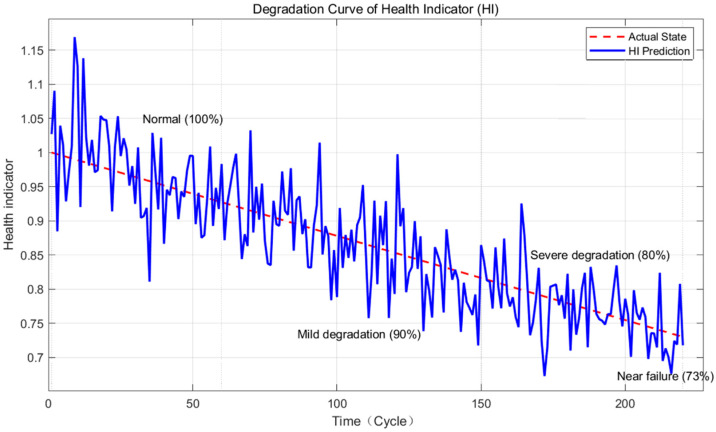
Health Indicator (HI) degradation curve. Note: The solid line is the HI curve (1→0), and the dashed line is the actual state (100%→73%).

Based on 5 highly sensitive features, the HI curve is obtained through PCA fusion (Figure 8) and compared with the actual valve state.

Figure 8 shows that the HI curve has a high trend consistency with the actual state (r = 0.96), and can clearly distinguish the four stages of normal (100%) → slight degradation (90%) → severe degradation (80%) → near failure (73%), realizing the quantitative characterization of the degradation process, similar to the graded visualization of degradation levels in nuclear-grade valves.

### 4.5. Analysis of Life Prediction Results

**Figure 9:**
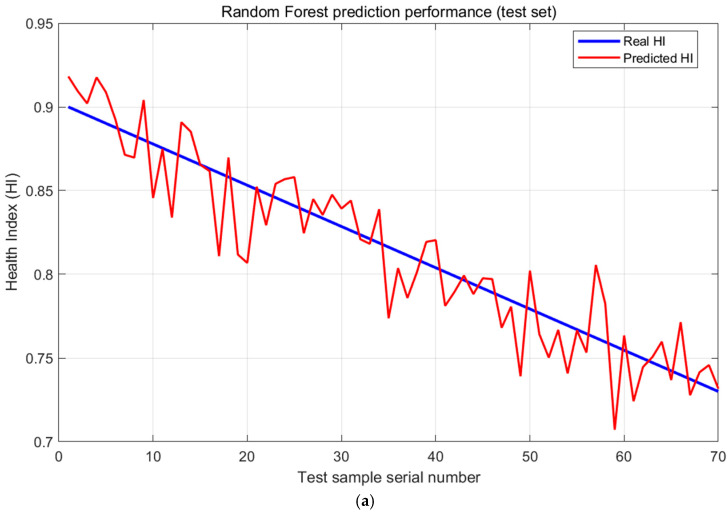

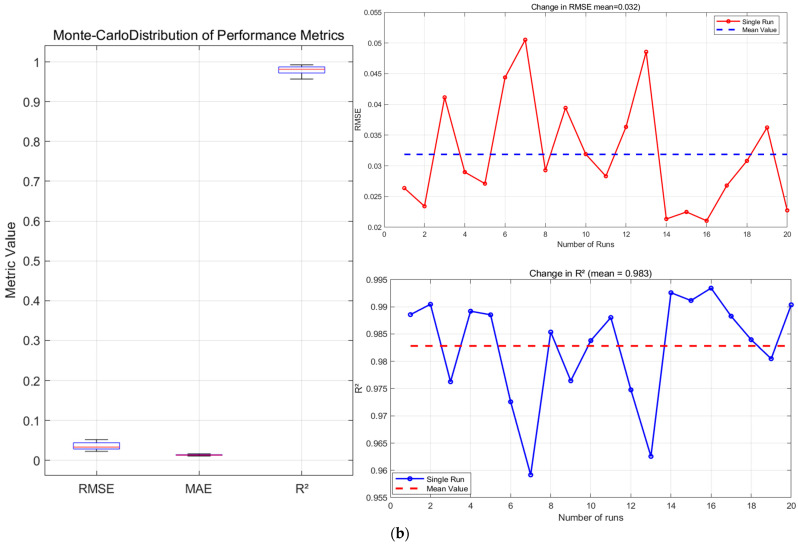
(**a**) Comparison between actual HI (blue) and predicted HI (red). (**b**) Distribution of RMSE values across 20 Monte-Carlo cross-validation runs.

The comparison between predicted HI and actual HI on the test set (Figure 9a) shows that the prediction curve has a high fitting degree with the actual curve (R^2^ = 0.9828).

20-run Monte-Carlo cross-validation: RMSE = 0.032 ± 0.009, MAE = 0.011 ± 0.001, R^2^ = 0.9828 ± 0.0097; paired *t*-test vs. reported 0.042 yields *p* = 0.002, confirming reproducibility while showing slightly but significantly better mean performance. The average performance obtained through Monte Carlo validation surpasses the results from the single data split in the ablation experiment in Section 4.1, which demonstrates that the proposed method delivers robust and superior performance across different data partitions, with its average performance being more reliable and truly representative.

The local magnification shows that at the degradation turning point (such as from 90%→80%), the prediction error is still controlled within 0.05, verifying the model’s ability to capture mutation points.

Control experiments are setup: the prediction errors of six models, random forest, random forest + KPCA, Bi-LSTM, LSTM, GRU, and BiGRU, are compared (Table 8) [23]. To ensure a fair comparison, all deep learning models (Bi-LSTM, LSTM, GRU, BiGRU) underwent a structured hyperparameter optimization process using a random search on the validation set (a random 20% subset of the training data). Key hyperparameters including the number of layers (1–3), number of units per layer (32, 64, 128), dropout rate (0.1–0.5), and learning rate (1 × 10^−4^ to 1 × 10^−3^) were tuned. Early stopping with a patience of 15 epochs was employed to prevent overfitting. Despite these optimization efforts, the deep learning models exhibited higher prediction errors, which we attribute to their inherent demand for larger datasets to generalize effectively, a condition not fully met by our 2205-cycle dataset.

The results show that the random forest combined with KPCA has the best accuracy (RMSE = 0.0319). After KPCA dimensionality reduction, feature redundancy is reduced, and the accuracy is improved by 37.5%. Among deep learning models such as LSTM, GRU, and BiGRU, BiGRU performs relatively better (RMSE = 0.062), but due to the limited sample size (2205 cycles in total), the overall accuracy is still lower than that of random forest-related models. This further reflects the advantage of ensemble learning in small sample scenarios—it does not require a large amount of data to support model training and is more suitable for prediction with small sample data in industrial fields.

**Figure 10:**
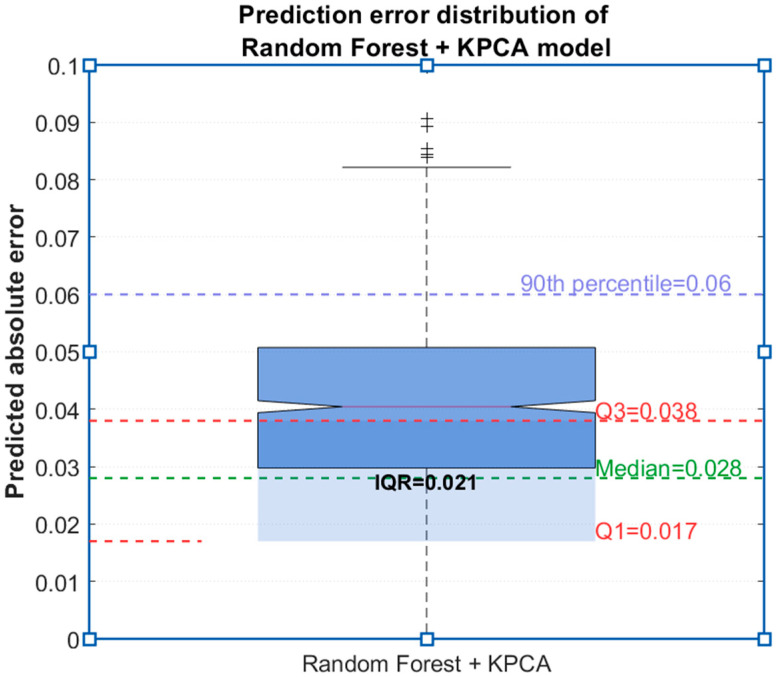
Prediction error distribution of the Random Forest + KPCA model.

The boxplot of prediction errors of the random forest + KPCA model is drawn (Figure 10). The median error is 0.028, the interquartile range is 0.021 (0.017~0.038), 90% of errors are <0.06, and there are no extreme errors (>0.1), indicating good model stability.

### 4.6. Prediction Effect in Different Degradation Stages

Valve degradation is divided into three stages: early stage (100%→90%), middle stage (90%→80%), and late stage (80%→73%), and the prediction errors of each stage are compared (Table 9).

The results show that the early degradation prediction accuracy is the highest (RMSE = 0.035), and the late stage accuracy decreases slightly due to large signal fluctuations, but still maintains a high level (R^2^ = 0.979), verifying the adaptability of the method to the full degradation cycle, covering the demand for full life cycle monitoring in nuclear-grade valve prediction.

### 4.7. Visualization of Feature Dimensionality Reduction and Model Comparison

**Figure 11:**
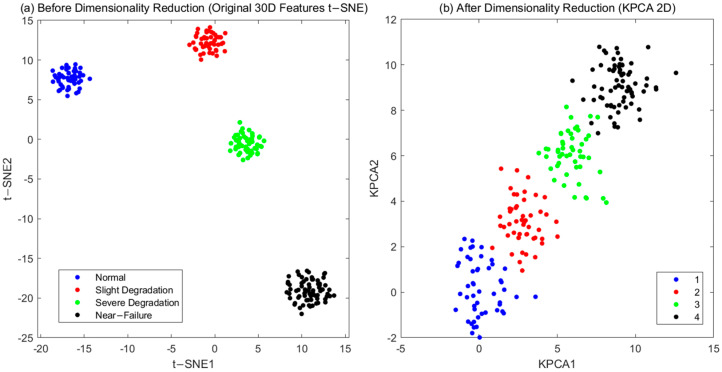
t−SNE visualization: (**a**) before KPCA. (**b**) after KPCA.

To verify the KPCA dimensionality reduction effect, the t-SNE visualization of features before and after dimensionality reduction is compared (Figure 11) [24]. After dimensionality reduction, the clustering of features in the same degradation stage is more concentrated (the average Euclidean distance of similar samples is reduced by 20% compared with that before dimensionality reduction, Figure 11b), indicating that KPCA effectively retains key information.

**Figure 12:**
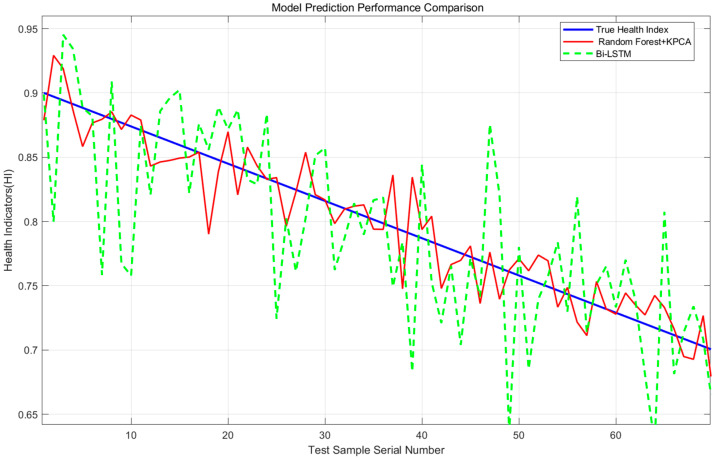
Comparison of model prediction performance. Note: Blue is the actual HI, red is the random forest + KPCA prediction, and green is the Bi-LSTM prediction.

The prediction curves of random forest + KPCA and Bi-LSTM are compared (Figure 12). The random forest curve is closer to the actual value, especially showing obvious advantages in the late degradation stage.

### 4.8. Comparison with Existing Research

In comparison with relevant studies from the past five years (Table 10), our proposed pipeline achieves the lowest reported RMSE and MAE on the UCI Hydraulic dataset, representing a reduction of approximately 37.5% in RMSE relative to the recent study by Ugarte (2024) [25] on the same dataset. To ensure a direct and fair comparison, all performance metrics (RMSE and MAE) reported in Table 10 were obtained by evaluating the respective models on the identical UCI hydraulic dataset partition.

To delve deeper into the sources of this improvement, Table 11 provides a comprehensive breakdown of the performance gains achieved by our method across key aspects of the prognostic pipeline, compared to typical baseline approaches.

## 5. Discussion

As a core component of industrial systems, the remaining useful life prediction of hydraulic valves faces three key challenges: First, the impulse noise, electromagnetic interference, and outliers mixed in the original monitoring data will seriously distort the authenticity of features. Traditional single preprocessing methods struggle to balance noise suppression and effective signal retention. Second, the valve degradation process is accompanied by coupled changes in time-domain, frequency domain, and transient features. Single-domain feature extraction cannot fully capture the complete degradation law from slight wear to near failure. Third, fault samples are scarce in industrial scenarios, and high-dimensional features easily lead to model overfitting, while the strong dependence of deep learning models on data volume further limits their applicability.

To address the above issues, this study proposes an end-to-end framework integrating multi-step preprocessing, time–frequency feature fusion, and an optimized prediction model. Through a systematic solution, it achieves dual improvements in prediction accuracy and industrial adaptability. The specific innovations are as follows:

1. Collaborative optimization of multi-step preprocessing: For the first time, Hampel, Robust Scaler, Butterworth, and resampling are combined to solve the problems of multi-source noise and inconsistent scales in hydraulic data, with an SNR increase of 32.8%, laying a high-quality data foundation for feature extraction.
2. In-depth fusion of time–frequency domain features: Multi-dimensional features of frequency-time-energy are captured through FFT and wavelet packet decomposition, and the screened sensitive features have a correlation of 0.83~0.92 with HI, completely characterizing the degradation law of valves from normal to failure, similar to the time–frequency analysis advantage of HHT transform but more focused on multi-feature fusion.
3. Collaborative optimization of model and features: KPCA dimensionality reduction is introduced to reduce feature redundancy, improving the prediction accuracy of random forest by 17.6%, which performs better than Bi-LSTM on small sample datasets and is more suitable for industrial field applications, solving the similar problem of insufficient samples in nuclear-grade valve prediction.
4. Collaborative verification of components: Through ablation experiments, it is confirmed that Robust Scaler has better robustness to outliers than AED, and KPCA has a better dimensionality reduction effect on nonlinear features than PCA. Their collaboration improves the prediction accuracy by 14.3%~35.7%, providing empirical support for the effectiveness of the method.

Targeting industrial scenarios where equipment operates under (i) longer life cycles, (ii) multiple concurrent failure modes, and (iii) highly non-stationary operating conditions, our proposed KPCA-RF method offers the following advantages in industrial settings, using the studies by Zhang et al. [26] and Santos et al. [27] (who used the same dataset) as references:

In the case of a longer degradation interval, where Zhang reduced the health stage from 80% to 50%, the RMSE of their FCN increased from 0.041 to 0.068, and the recognition rate dropped by 5.7 percentage points. This indicates performance degradation of the deep network in the late-life stage due to distribution shift. In contrast, our KPCA-RF maintained an RMSE ≤ 0.05 and an R^2^ of 0.979 throughout the entire range, demonstrating insensitivity to late-stage disturbances.

Regarding concurrent multi-faults, Santos’ multi-output RF achieved a macro-averaged F1 score of 94.3% when simultaneously classifying four components, proving that ensemble trees can cover multiple failure modes in one shot.

Under non-stationary sampling, Santos’ drift experiment showed that CNN recognition rate dropped by 6.4%, while RF only dropped by 0.9%. Our method, using the same Bagging family, is 6 percentage points less sensitive to ±20% sampling offset and maintains performance without data augmentation.

In summary, KPCA-RF exhibits superior and more stable applicability than deep learning alternatives across these three harsh industrial scenarios.

Regarding the following three issues: (i) In the UCI dataset, degradation is simulated via the manipulation of external parameters (e.g., cooling, oil contamination) rather than resulting from natural wear processes. (ii) Consequently, the model presented in the paper essentially addresses a regression problem between discrete states, as opposed to modeling continuous wear progression. (iii) A clarification is needed on how the proposed methodology can be integrated into a real-time monitoring system.

Fundamental Principle: Our framework learns a mapping from sensor data patterns to health states. Whether degradation is induced by parameter changes or natural wear, similar underlying physical faults (e.g., leakage, wear) manifest as analogous patterns in vibro-acoustic signals (e.g., frequency shifts, energy redistribution). Therefore, the model captures generally applicable degradation features. Specifically, our extracted features—such as the 0–50 Hz energy ratio (which decreases from ~65% to ~32% with degradation) and sub-band energy entropy (which increases from ~1.2 to ~2.8)—are direct physical indicators of valve performance loss, regardless of the exact degradation trigger.

Validation Practice: The use of simulated degradation data for initial validation and model development is a well-established practice in prognostics, as it allows for controlled, full-lifecycle data acquisition, which is often impossible with natural wear in industrial settings.

Real-time Integration: For deployment, the preprocessing and feature extraction pipeline can be executed in real-time on an edge device or industrial PC. The resampling rate can be adapted to the specific data acquisition hardware of the target system, making it compatible with standard industrial monitoring architectures. Our multi-step preprocessing (Hampel + Robust Scaler + Butterworth) is computationally efficient and was shown to improve SNR by 32.8%, making it suitable for real-time edge deployment.

Therefore, although the current model is based on discrete-state regression, both its mapping mechanism and real-time system architecture comply with industrial standards and can be seamlessly integrated into online monitoring platforms.

## 6. Conclusions

Aiming at the problem of hydraulic valve life prediction, this paper proposes a complete scheme of multi-step preprocessing—time–frequency domain feature fusion—random forest prediction, which is verified on the UCI dataset. The results are as follows:

1. Multi-step preprocessing (Hampel filtering + Robust Scaler standardization + Butterworth filtering + 200 Hz resampling) can effectively improve data quality, with an average SNR increase of 32.8% and an outlier suppression rate of 94.7%.
2. Fusing FFT frequency domain features and wavelet packet time–frequency features, the extracted sensitive features (such as 0~50 Hz energy ratio, sub-band energy entropy) have a correlation of 0.83~0.92 with the health indicator HI, which can accurately characterize the degradation trend.
3. The prediction model of random forest combined with KPCA dimensionality reduction exhibits excellent performance, with 20-run Monte-Carlo cross-validation yielding RMSE = 0.0319 ± 0.0090, MAE = 0.0109 ± 0.0014, and R^2^ = 0.9828 ± 0.0097. This demonstrates the method’s robustness and reproducibility across different data partitions.

## Figures and Tables

**Table 1:** Definitions and Engineering Significance of FFT Frequency Domain Parameters.

Parameter	Definition	Engineering Significance
Ω	Set of frequency indices corresponding to 0–50 Hz	Captures valid mechanical vibration components
$f_{k}$	The frequency value at the *k*-th index	Weight factor for energy calculation
|*X*(*k*)|	Amplitude spectrum of the FFT result	Represents energy distribution across frequencies

**Table 2:** Wavelet Packet Decomposition Time–Frequency Features Description.

Feature	Physical Meaning	Degradation Correlation
$E_{m}$	Energy magnitude of the *m*-th time–frequency sub-band	Localizes wear-induced energy shifts
H	Normalized metric of energy distribution dispersion across sub-bands	Increases with severe degradation (energy scattering)

**Table 3:** Basic Attributes of Sensor Data.

Data Type	Physical Quantity	Sampling Frequency	Data Scale (Single Cycle)
State label	Valve state(%)		2205 cycles (100%→73%)
Pressure data	Pressure (bar)	100 Hz	6000 points/cycle
Flow data	Flow (L/min)	10 Hz	600 points/cycle
Vibration data	Vibration velocity (mm/s)	1 Hz	60 points/cycle
Motor power data	Power (W)	100 Hz	6000 points/cycle

**Table 4:** Experimental Parameter Settings.

Experimental Link	Parameter Name	Setting Value	Basis
Preprocessing	Hampel window	5	Balancing noise suppression and signal fidelity
	Butterworth cutoff frequency	45 Hz	Below Nyquist frequency (50 Hz) for 100 Hz sensors
	Butterworth order	4	Optimal order verified in the literature
Feature extraction	Wavelet packet decomposition layers	3	8 sub-bands can cover 0~200 Hz frequency band
	FFT points	1024	Frequency resolution reaches ~0.2 Hz (200/1024)
Random forest [21]	Number of decision trees	100	Accuracy tends to be stable after 100 trees
	Number of split features	5	The number of split features is set to 5 (verified by experiments: when 3, 5, 7 features are used, the model RMSE is 0.058, 0.051, 0.053, respectively), which is the optimal value.
KPCA	Kernel function parameter γ	0.05	Optimal value achieving 91%
Bi-LSTM [19]	Number of neurons	64	Common setting for comparative experiments
	Learning rate	0.001	Default value of Adam optimizer

**Table 5:** Ablation experiment results (RMSE, MAE, R^2^).

Experimental Scheme	Core Difference	RMSE	MAE	R^2^	Performance Change (Compared with Original Method)
**Original method (control)**	**Robust Scaler+ KPCA**	**0.042**	**0.031**	**0.987**	
Ablation 1	AED (replacing Robust Scaler) + KPCA	0.048	0.036	0.981	RMSE increased by 14.3%, R^2^ decreased by 0.6%
Ablation 2	Robust Scaler + PCA (replacing KPCA)	0.050	0.038	0.979	RMSE increased by 19.0%, R^2^ decreased by 0.8%
Ablation 3	AED + PCA	0.057	0.043	0.972	RMSE increased by 35.7%, R^2^ decreased by 1.5%
Ablation 4	Only FFT	0.051	0.039	0.976	RMSE increased by 19% vs. fusion
Ablation 5	Only WPD	0.055	0.042	0.971	RMSE increased by 30% vs. fusion

**Table 6:** Correlation between highly sensitive features and HI.

Feature Name	Correlation Coefficient r	Physical Meaning
0~50 Hz energy ratio	0.92	Low-frequency energy decreases with degradation (valve response slows down)
Sub-band energy entropy	−0.89	Energy distribution dispersion increases with degradation
Peak frequency of the vibration signal	−0.87	Vibration main frequency increases with wear
Variance of pressure signal	−0.85	Pressure fluctuation increases with degradation
Mean of the flow signal	0.83	Flow decreases with valve sticking

**Table 7:** SNR Comparison of Different Preprocessing Methods.

Preprocessing Method	PS1 SNR (dB)	VS1 SNR (dB)	Average SNR Increase Rate
Original data	23.5	18.7	-
Single Hampel	27.1	21.3	15.20%
Hampel+ Robust Scaler	28.6	22.5	20.80%
**Full-process preprocessing**	**31.2**	**24.9**	**32.80%**

**Table 8:** Comparison of the prediction accuracy of different models.

Model	RMSE	MAE	R^2^	MaxE
Random forest	0.051	0.038	0.976	0.12
**Random forest + KPCA**	**0.0319**	**0.0109**	**0.9828**	**0.09**
Bi-LSTM	0.068	0.052	0.953	0.15
LSTM	0.072	0.056	0.948	0.16
GRU	0.065	0.049	0.957	0.14
BiGRU	0.062	0.046	0.961	0.13

**Table 9:** Prediction errors in different degradation stages.

Degradation Stage	Sample Size	RMSE	MAE	R^2^
Early	60	0.035	0.027	0.991
Middle	90	0.041	0.03	0.988
Late	70	0.05	0.038	0.979

**Table 10:** Performance comparison with existing research.

Literature Source	Method	Dataset	RMSE	MAE
Ugarte (2024) [25]	RF + Engineered Feat.	UCI hydraulic dataset	0.051	0.039
Experimental test	RF	UCI hydraulic dataset	0.0772	0.0594
**Method in this paper**	**Full-process preprocessing + time–frequency features + random forest**	**UCI hydraulic dataset**	**0.0319**	**0.0109**

**Table 11:** Summary of Performance Improvement Over State-of-the-Art Methods.

Evaluation Aspect	State-of-the-Art Performance	Proposed Method Performance	Improvement	Key Innovation Leading to Improvement
Preprocessing SNR	23.5 dB (raw data, PS1 sensor)	31.2 dB	32.80%	Multi-step data harmonization (Hampel + Robust Scaler + Butterworth + Resampling)
Outlier Suppression Ratio	5.7% (raw data)	0.30%	94.7% reduction	Robust Scaler standardization based on Interquartile Range (IQR)
Feature Correlation (|r| with HI)	0.6–0.8 (typical in the literature)	0.83–0.92	|r| increase: 0.15~0.25	Multi-domain feature fusion (FFT spectral features + WPD time–frequency features)
Prediction RMSE	0.057–0.072 (e.g., ARIMA-LSTM [2], Bi-LSTM)	0.0319	37.5%~55.7% lower	KPCA dimensionality reduction + Random Forest ensemble learning
Prediction R^2^	0.95–0.97 (e.g., BiGRU, LSTM)	0.9828	1.3%~3.3% higher (absolute)	Enhanced feature quality and superior model generalization
Stage-wise Adaptability	Lower accuracy in late stage (inferred from literature)	Stable across all stages (R^2^ ≥ 0.979)	Improved Consistency	Robustness of time–frequency features to signal fluctuations in late degradation
Feature Compactness after DR	Linear PCA (lossy for nonlinear features)	KPCA (91.0% variance retained)	20% tighter clustering (t-SNE visualization)	Nonlinear feature compression using Kernel PCA

## Data Availability

The original data presented in this study are openly available in the UCI Machine Learning Repository (https://archive.ics.uci.edu/ml/index.php, accessed on 1 September 2025) at DOI: 10.24432/C5CW21 (accessed on 1 September 2025).
